# Lignocellulose Nanofibre Obtained from Agricultural Wastes of Tomato, Pepper and Eggplants Improves the Performance of Films of Polyvinyl Alcohol (PVA) for Food Packaging

**DOI:** 10.3390/foods10123043

**Published:** 2021-12-08

**Authors:** Isabel Bascón-Villegas, Mónica Sánchez-Gutiérrez, Fernando Pérez-Rodríguez, Eduardo Espinosa, Alejandro Rodríguez

**Affiliations:** 1 Department of Food Science and Technology, Faculty of Veterinary, Agrifood Campus of International Excellence (ceiA3), University of Cordoba, 14014 Córdoba, Spain; q12bavii@gmail.com (I.B.-V.); v02sagum@uco.es (M.S.-G.); 2 BioPrEn Group (RNM940), Inorganic Chemistry and Chemical Engineering Department, Faculty of Science, Agrifood Campus of International Excellence (ceiA3), University of Cordoba, 14014 Córdoba, Spain; a02esvie@uco.es (E.E.); q42ropaa@uco.es (A.R.)

**Keywords:** plastic package, biorefinery, agri-food by-products, valorisation, films

## Abstract

Films formulated with polyvinyl alcohol (PVA) (synthetic biopolymer) were reinforced with lignocellulose nanofibres (LCNF) from residues of vegetable production (natural biopolymer). The LCNF were obtained by mechanical and chemical pre-treatment by 2,2,6,6-tetramethylpiperidine-1-oxyl radical (TEMPO) and added to the polyvinyl alcohol (polymer matrix) with the aim of improving the properties of the film for use in food packaging. The mechanical properties, crystallinity, thermal resistance, chemical structure, antioxidant activity, water barrier properties and optical properties (transparency and UV barrier), were evaluated. In general, with the addition of LCNF, an improvement in the studied properties of the films was observed. In terms of mechanical properties, the films reinforced with 7% LCNF TEMPO showed the best results for tensile strength, Young’s modulus and elongation at break. At the same LCNF proportion, the thermal stability (T_max_) increased between 5.5% and 10.8%, and the antioxidant activity increased between 90.9% and 191.8%, depending on the raw material and the pre-treatment used to obtain the different LCNF. Finally, a large increase in UV blocking was also observed with the addition of 7% LCNF. In particular, the films with 7% of eggplant LCNF showed higher performance for Young’s modulus, elongation at break, thermal stability and UV barrier. Overall, results demonstrated that the use of LCNF generated from agricultural residues represents a suitable bioeconomy approach able to enhance film properties for its application in the development of more sustainable and eco-friendly food packaging systems.

## 1. Introduction

Food packaging plays a major role in protecting, maintaining and improving the quality and safety of food during transport, distribution and storage [1]. Conventionally, the materials used to produce food packaging have been plastic polymers of petrochemical origin, (polyethylene terephthalate (PET), polyvinyl chloride (PVC), polyamide (PA), polystyrene (PS), polypropylene (PP), polyethylene (PE), etc.), paper, metal and glass. The widespread use of plastic packaging is due to their mechanical properties, rigidity and flexibility, barrier properties against oxygen and moisture, low cost and ease of production [2,3]. In 2018, plastics production reached 51.2 million tonnes, accounting for 39.9% of food packaging production [4].

Despite the technological advantages of using plastic packaging, environmental and human health can be negatively affected due to its non-biodegradable nature and the possibility of migration of chemicals from plastic packaging into food [2]. For this reason, there is an increasing interest in searching for a suitable substitute of such synthetic polymers [5]. The development and use of bio-based, abundant and sustainable resources is proposed as one of the alternatives to conventional materials [6].

The origin of biopolymers can be natural (starch, chitosan, cellulose, etc.), and synthetic (polylactic acid (PLA), polyglycolic acid (PGA), polyvinyl alcohol (PVA), etc.) [7]. Synthetic biopolymers present potential to improve packaging properties such as durability, biodegradability, flexibility and high brightness [8]. Despite these advantages, these materials have poorer mechanical, thermal and barrier properties compared to conventional plastic materials. With the development of nanotechnology, several nanofillers have arisen for application in food packaging materials. Among them, nanocellulose have shown excellent performance that when added into the polymer, can improve the mechanical and barrier properties of the synthetic biopolymer [9].

PVA is a biodegradable polymer with very good chemical resistance, high crystallinity, good film formation, hydrophilic properties and no toxicity [10]. This biopolymer has great potential as food packaging due to its excellent tensile strength, flexibility and oxygen barrier properties [11]. However, due to the presence of a large amount of hydroxyl groups, they have a hydrophilic nature, which in wet environments causes the penetration of water molecules through the film, leading to a deterioration in the mechanical properties and oxygen barrier properties [12]. Therefore, the use of PVA as food packaging would be restricted to food products associated with low relative humidity. Nonetheless, the addition of cross-linker compounds (dialdehydes, dicarboxylic acids, dianhydrides) may decrease the hydrophilic behaviour of the material, making it more suitable for high water content environments [13,14,15].

Cellulose nanofibres (CNF) play an effective role in polymer reinforcement [16]. This reinforcement is due to the presence of aromatic hydroxyl groups, which through hydrogen bonding, lead to a good interaction between the polymeric matrices [17,18,19]. These CNF could be obtained from plant biomass proceeding from agricultural by-products, making it a highly available and very low-cost resource. The CNFs could be used for the manufacture of food packaging, resulting in value-added materials produced in a sustainable and environmentally friendly way [20]. In 2020, vegetable production in Spain reached almost 16 million tonnes, with Andalusia being the region with the highest vegetable production (33% of the total) [21]. This high production leads to the generation of a large amount of agricultural waste that needs to be managed. An alternative way of managing agricultural waste would be its use in the production of value-added materials, avoiding its traditional applications (burning, composting, animal feed, etc.), thus enhancing the circular economy system [22]. In a previous study of our laboratory, lignocellulose nanofibres (LCNF) were satisfactorily produced from woody residues from tomato, pepper and eggplant and characterised for physicochemical and antioxidant properties [23].

The aim of this study was the development of films for food application, assessing the effect of pre-treatment and concentration of lignocellulose nanofibres (LCNF) from woody residues from tomato, pepper and eggplant plants in a polymeric matrix (PVA). The effect on the mechanical properties (tensile strength, Young’s modulus and elongation at break), crystallinity (XRD), thermal resistance (TGA), chemical structure (FTIR), antioxidant activity (ABTS assay), barrier properties (WVP) and optical properties (transparency and UV barrier) was studied in the developed films.

## 2. Materials and Methods

### 2.1. Reagents

The woody wastes used for this study, tomato (*Solanum lycopersicum*), pepper (*Capsicum annuum*) and eggplant (*Solanum melongena*) crops were provided by Cooperativa Aguadulce (Almería, Spain). The material was cleaned and dried at room temperature prior to cutting (0.1–1 cm) and stored in plastic bags.

The polymeric matrix, polyvinyl alcohol (P.M.: 146,000–186,000, and hydrolysis degree +99%) was supplied by Sigma Aldrich, Spain. Films were generated and characterized using different methods with the use of sodium hydroxide (Panreac, Germany), sodium bromide (Honeywell, NC, USA), TEMPO (2,2,6,6-Tetramethyl-piperidin-1-oxyle) (Sigma Aldrich, Madrid, Spain), sodium hypochlorite (Panreac, Castellar del Vallès, Barcelona, Spain), ABTS (2,2′-Azinobis-(3-ethylbenzthiazoline-6-sulphonate acid) (Sigma Aldrich, Madrid, Spain), calcium chloride anhydrous (Scharlab, Barcelona, Spain).

### 2.2. Cellulose Pulp Obtention

The raw materials were subjected to an environmentally friendly soda pulping process, in a 15 L batch reactor with a liquid–solid ratio of 10:1, 7% NaOH on dry material (o.d.m) at 100 °C for 150 min. The cellulosic pulp was disintegrated at 1200 rpm for 30 min and passed through a Sprout-Bauer refiner. The resulting pulp was sieved through a 0.14 mm mesh to retain uncooked material [23]. To remove excess water from the pulp, it was centrifuged and allowed to dry at room temperature.

### 2.3. Lignocellulose Nanofibres Production

Cellulose pulp was submitted to mechanical and TEMPO-mediated pre-treatments, followed by a high-pressure homogenisation treatment to obtain lignocellulose nanofibres [24].

#### 2.3.1. Mechanical Pre-Treatment

The cellulose pulp obtained was beaten with a PFI beater until the degree of drainage (°SR) closest to 90° was obtained. All samples required 30,000 revolutions to obtain the highest SR value, according to ISO 5264-2:2002.

#### 2.3.2. TEMPO-Mediated Oxidation Pre-Treatment

The cellulose pulp was subjected to mechanical beating in a PFI beater (4000 revolutions), prior to chemical treatment. Then, a suspension of NaClO (12%) (5 mmol sodium hypochlorite) was added to the obtained pulp at room temperature with continuous stirring. To stabilise and maintain the pH of 10.2, NaOH (0.5 M) was added. Finally, the fibres were filtered and washed several times with distilled water [25].

#### 2.3.3. High-Pressure Homogenization

The pre-treated fibre suspensions (1% *w*/*v*) were homogenised at high pressure with a Panda GEA 2 K NIRO homogeniser (GEA, Düsseldorf, Germany). This process was carried out in 10 cycles (4 cycles at 300 bar, 3 cycles at 600 bar and 3 cycles at 900 bar) [26].

### 2.4. Film Formulation and Production

The PVA solution (3 wt.%) was prepared in distilled water at 90 °C for 4 h with constant mechanical stirring. The different LCNF obtained at various concentrations of 2, 5 and 7% (*w*/*w*) were added to the polymer suspension (Table 1). The mixtures were stirred at room temperature for 4 h. The resulting suspensions were poured into polypropylene Petri dishes (ø = 9 cm) and allowed to dry at room temperature. The total dry weight of the mixture was 0.35 g per slide.

### 2.5. Film Characterisation

#### 2.5.1. Mechanical Properties

The analysis of the mechanical properties of films is relevant to determine their strength and durability [27].

Prior to the measurements, the films with size (65 × 15 mm) were conditioned for 48 h at 25 °C and 50% relative humidity. The thickness of the films was measured using a Digital Micrometre IP65 0–1”, Digimatic, Mitutoyo (Neuss, Germany). The ASTM D638 test method was followed to determine the tensile strength, Young’s modulus and elongation at break of PVA and LCNF-PVA films. A Lloyd Instrument LF Plus testing machine (AMETEK Measurement & Calibration Technologies Division, Largo, FL, USA) equipped with a 1 kN load cell was used applying a speed of 100 mm/min. Ten replicates of each sample were measured.

#### 2.5.2. X-ray Diffraction (XRD) Analysis

XRD was used to assess and characterize the crystal structure of films formulated, and the changes observed with the addition of LCNF to the polymeric matrix. This analysis was performed using a Bruker D8 Discover with a monochromatic source CuKα1 over an angular range of 5–50° at a scan speed of 1.56°/min.

#### 2.5.3. Thermogravimetric Analysis (TGA)

TGA was performed to evaluate the thermal stability of the different films obtained. In addition, the maximum degradation rate (T_max_) was calculated from the thermogravimetric derivative (DTG). The analysis was performed with a Mettler Toledo TGA/DSC 1 (Mettler Toledo, L’Hospitalet de Llobregat, Barcelona, Spain). The samples were brought from room temperature to 600 °C with a heating rate of 10 °C/min under nitrogen atmosphere (50 mL/min gas flow).

#### 2.5.4. Spectroscopy Analysis

To compare the changes generated in the polymer matrix by the addition of different concentrations of LCNF, the FTIR spectra of each of the films obtained were analysed. For that end, FTIR spectra were obtained using the FTIR-ATR PerkinElmer Spectrum Two (Waltham, MA, USA) with a resolution of 4 cm^−1^ in the range 450–4000 cm^−1^, performing 40 scans per sample.

#### 2.5.5. Antioxidant Activity

The evaluation of the antioxidant capacity or power of the films was performed by the ABTS (2,2′-Azinobis-(3-ethylbenzthiazoline-6-sulphonate acid) assay.) For the preparation of the ABTS solution (7 mM), 38 mg of the ABTS reagent was diluted in 10 mL of potassium persulphate (2.45 mM) and kept in the dark for 16 h. Then, an ABTS–ethanol solution (50% *v*/*v*) was prepared to obtain an absorbance of 0.70 ± 0.02 nm at 734 nm [28]. For the sample measurement, 5 mg of sample with 2 mL of ABTS–ethanol solution was placed in the measuring cuvette, shaken and allowed to stand for 6 min before measurement at 734 nm.

The following equation was used to determine the antioxidant capacity

AOP = (A_ABTS6´_ − A_ABTSfilm6´_/A_ABTS0´_) × 100 (1)

where A_ABTS6´_ is the absorbance at 734 nm of the radical solution after 6 min; A_ABTSfilm6´_ is the absorbance at 734 nm of the sample after 6 min; A_ABTS0´_ is the absorbance at 734 nm of the radical solution before 6 min. ABTS scavenging activity was expressed as %AOP per mg of the film. All assays were performed in triplicate.

#### 2.5.6. Water Permeability

The provision of a barrier between the external environment and the food is a fundamental requirement in the development of films for food use. This film must protect the food, and, in this respect, water vapour permeability is very relevant due to the role of moisture plays in the shelf life of food [29,30].

The standard test method for water vapour transmission of materials (ASTM E96/E96M-10) was used for this analysis. Periodic weighing was carried out to monitor the mass increase of the sample, produced by the passage of water vapour through the film into the desiccant material consisting of calcium chloride anhydrous. The test was performed at 25 °C and 50% relative humidity.

To calculate the water vapor permeability (WVP) the following formula was used

WVP = (WVTR·I)/(P_sat_ × (RH_out_ − RH_in_)) (2)

where WVTR is the water vapour transmission rate; I is the thickness; RH_out_ is the external relative humidity (50%) and RH_in_ is the internal relative humidity (0%); P_sat_ is the saturation vapor pressure at the test temperature.

#### 2.5.7. Optical Properties

The light transmittance of the film in the UV-VIS regions (200–800 nm) was determined with a PerkinElmer UV/VIS Lambda 25 spectrophotometer (Waltham, MA, USA).

The following equations were used to determine the transparency and UV-barrier properties

Transparency = log %T_660_/I (3)

where %T_660_ is the percent transmittance at 660 nm, and I is the film thickness (mm).

UV−barrier = 100 − (%T_280_/%T_660_) × 100 (4)

where %T_280_ is the percent transmittance at 280 nm, and %T_660_ is the percent transmittance at 660 nm.

#### 2.5.8. Statistical Analysis

To evaluate the influence of raw material, pre-treatment and LCNF concentration on film formulation, Univariate Analysis of Variance (ANOVA) and *t*-test for independent samples were carried out using the IBM^®^ SPSS^®^ version 25 statistical software (IBM Corporation, New York, NY, USA).

## 3. Results

### 3.1. Mechanical Properties

**Figure 1:**
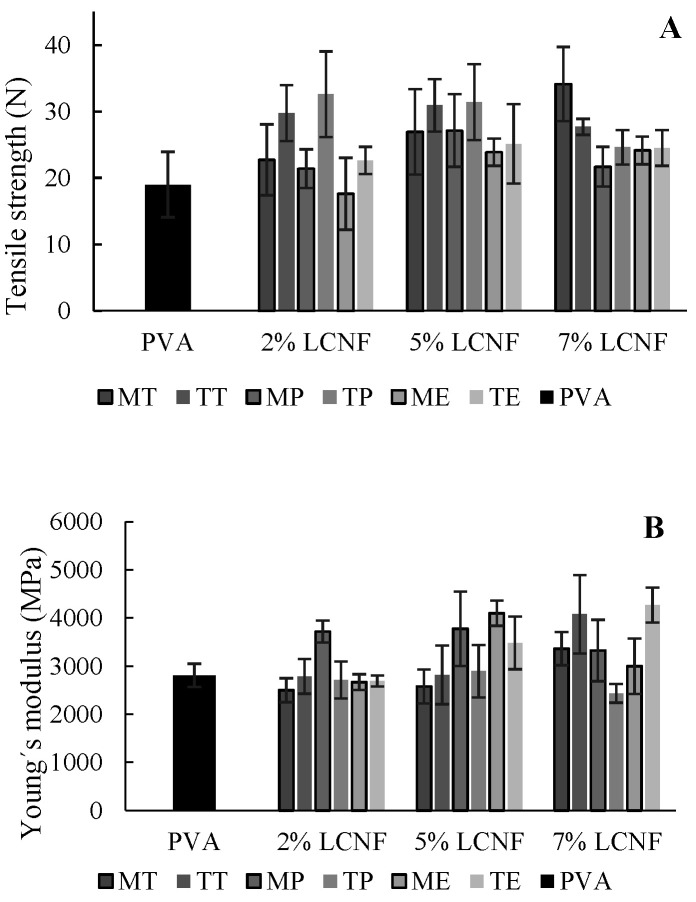

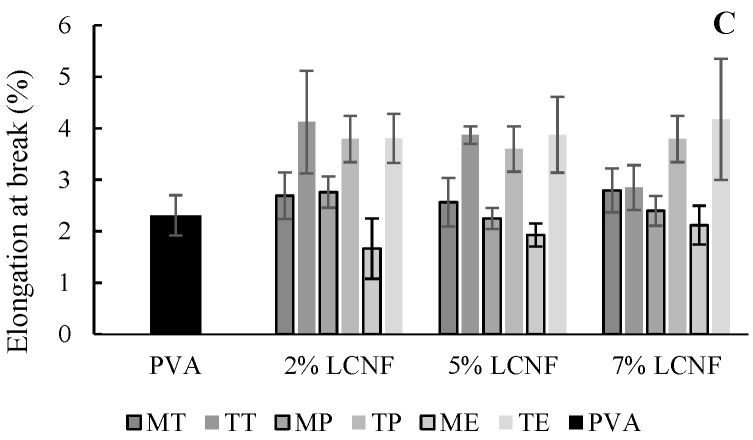
Mechanical properties of PVA film (**A**), PVA films containing mechanical LCNF from tomato (MT), pepper (MP) and eggplant (ME) (**B**), and PVA films containing TEMPO LCNF from tomato (TT), pepper (TP) and eggplant (TE) (**C**).

Figure 1 shows the effect of the addition of different proportions of LCNF in the polymer matrix on the mechanical properties of the films. According to the results, mechanical properties could be significantly improved for some of the tested conditions. The better performance observed could be due to different mechanisms: (i) the compact structure of PVA, (ii) the stiffness of the LCNF chain, (iii) the homogeneous distribution of LCNF in the polymeric matrix and (iv) the strong interaction between the OH groups of LCNF and PVA [31]. 

In the tensile strength, a significant increase was reported for samples with LCNF from tomato and pepper (*p* < 0.05) compared to the PVA films. In this sense, a decrease in tensile strength was observed for most of the samples when increasing the LCNF concentration from 5% to 7%, except for MT-LCNF and ME-LCNF. This slight decrease in film tensile strength at higher LCNF concentration may be due to the rate of LCNF agglomeration in films formulated with PVA. This agglomeration may lead to a breakdown of the interaction of the two matrices and result in weaker areas in the film [27,32]. The TEMPO pre-treatment applied to obtain the LCNF also showed an improvement for this property, except for the 7% TT-LCNF sample.

For Young´s modulus, the addition of 7% LCNF led to a remarkable rise in this parameter, although it was only apparent for tomato and eggplant (*p* < 0.05). However, overall, raw material and pre-treatment had no significant effect on the improvement of this property.

The elongation at break of the films was statistically higher in TEMPO-treated samples (*p* < 0.05) excepting for tomato, in which at 7% LCNF, values were similar. The highest increase (80%) was found in 7% TE-LCNF samples. This improvement with the addition of TEMPO LCNF could be due to the partial loss of lignin from the fibres due to oxidation, which leads to stronger hydrogen bonds, resulting in an improvement in this property. This partial loss of lignin can be seen in Figure S1. Besides that, the raw material used and the concentration of LCNF added to the polymeric matrix did not have a statistically significant effect (*p* > 0.05) on the improvement of this property.

### 3.2. X-ray Diffraction Analysis

**Figure 2:**
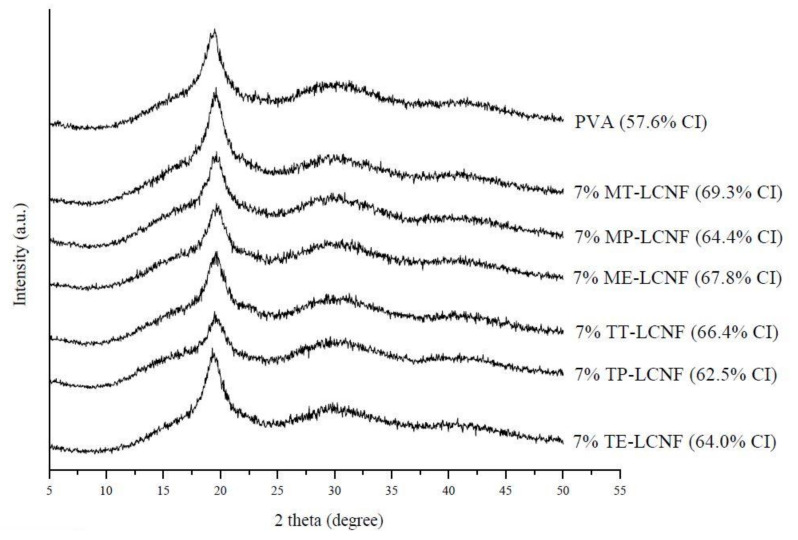
X-ray diffractograms of PVA film, PVA films containing 7% mechanical LCNF from tomato (MT), pepper (MP) and eggplant (ME), and PVA films containing 7% TEMPO LCNF from tomato (TT), pepper (TP) and eggplant (TE).

Figure 2 shows the X-ray diffraction patterns of the PVA films and the PVA films with 7% of different LCNFs. The PVA film showed the typical strong semi-crystalline structure typical of such a polymer. The diffractogram shows the highest intensity peak at 19.7°, due to the hydrogen bonds between the hydroxyl groups of the PVA chains [33]. The diffractograms obtained from the films with 7% LCNF showed higher intensity signals between 19.45° and 19.71°, very similar to the PVA films, therefore, the addition of LCNF to the polymeric matrix did not modify the semi-crystalline structure of PVA. The typical signals associated with cellulose: 14.7° plane (101), 20.4° plane (102) and 22.7° plane (200) [34] were very dimly observed in the diffractogram.

### 3.3. Thermogravimetric Analysis

Thermal stability is one of the most important properties because of its influence on other properties such as mechanical strength, durability and shelf life. Thermal degradation can lead to these properties gradually deteriorating [35].

**Figure 3:**
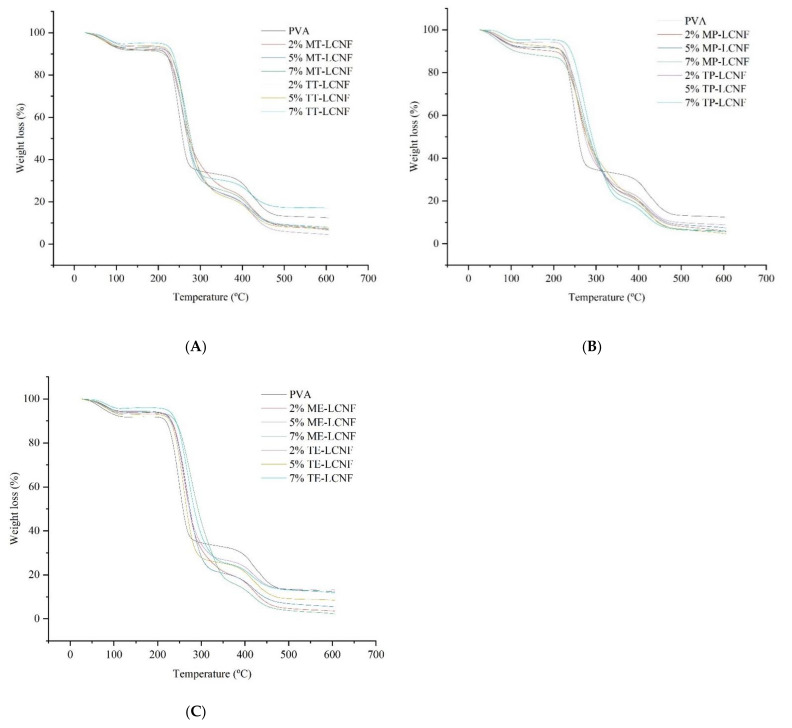
Thermogravimetry analysis (TGA) of PVA films, PVA films containing mechanical and PVA films containing TEMPO LCNF from tomato (MT, TM) (**A**), pepper (MP, TP) (**B**) and eggplant (ME, TE) (**C**).

The thermal stability of PVA films and PVA–LCNF films was assessed by calculating degradation onset temperature (T_onset_) and the maximum degradation temperature (T_max_) using the derivative of the TGA (DTG). Three stages of degradation were observed as shown in Figure 3, which were attributed to the decomposition of the PVA matrix as it is the major component of the film. The first stage (100 °C) corresponds with the water evaporation. The most pronounced losses occurred in the second (200–400 °C) and third stages (400–500 °C) due to the loss of the PVA structure [36].

Table 2 shows the T_onset_ and T_max_ of formulated films. The film formulated with only PVA had the lowest T_max_ (250.32 °C) of all formulated films. The highest T_max_ values were found in films formulated with 7% LCNF, with a maximum increase of 10.80% reached in 7% ME-LCNF samples, as compared to samples with only PVA (Table 2). This fact signals that the addition of LCNF in the polymer matrix improved its thermal resistance [37]. An effect of the raw material on T_max_ could be confirmed, which was more evident in formulations with mechanical LCNF and, particularly, was higher in samples with eggplant mechanical LCNF (Table 2). The higher thermal stability of mechanical samples may be because of a major presence of lignin as compared to TEMPO pre-treatment, which usually yields a partial loss of lignin content. Lignin establishes covalent bonds with cellulose, conferring a higher thermal stability [38,39].

### 3.4. Spectroscopy Analysis

**Figure 4:**
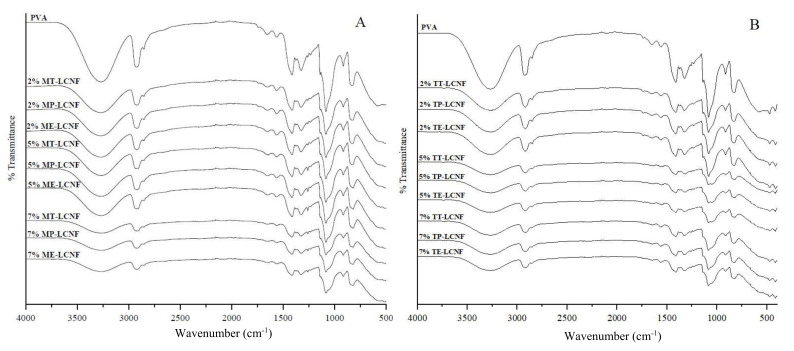
FTIR spectra of PVA films, PVA containing mechanical LCNF (**A**) from tomato (MT), pepper (MP) and eggplant (ME) and PVA films containing TEMPO LCNF (**B**) from tomato (TT), pepper (TP) and eggplant (TE).

Figure 4 shows the FTIR spectra of PVA and PVA–LCNF films with different percentages of LCNF. Both types of film showed a peak at 3259.6 cm^−1^, indicating free hydroxyl groups due to the strong intermolecular and intramolecular bonds. The peak at 2930 cm^−1^ corresponds to the C–H stretching vibrations of methyl or methylene groups. The peak detected at 1425 cm^−1^ corresponds with the bending mode of CH_2_ bonds. The C–O stretching was observed at 1330 cm^−1^. At 920 cm^−1^, a peak characteristic of the structure of CH_2_ groups was observed. The absorption band at 840 cm^−1^ correspond to the stretching vibration of the C–C groups [27,40,41,42,43]. Nonetheless, the FTIR analysis results did not indicate drastic changes in the chemical structure of PVA with the addition of LCNF. Other authors reported that the spectra obtained did not show any new chemical structures or chemical reactions between the components used for film formation [44]. In our study, the similar structure observed among formulations could be due to the low concentration of LCNF added to the PVA polymer matrix. Furthermore, no changes were observed in the spectra as a function of the pre-treatment applied to LCNF.

### 3.5. Antioxidant Activity

**Figure 5:**
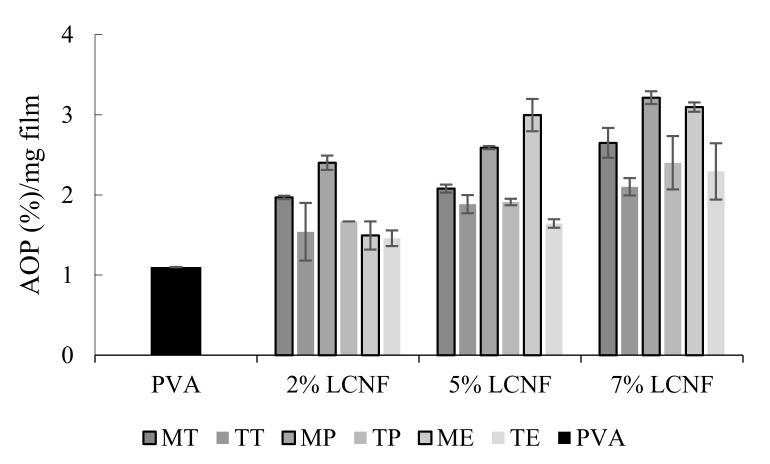
Antioxidant power (AOP) of PVA film, PVA films containing mechanical LCNF from tomato (MT), pepper (MP) and eggplant (ME) and PVA films containing TEMPO LCNF from tomato (TT), pepper (TP) and eggplant (TE).

Figure 5 shows the antioxidant activity of PVA films and PVA films with LCNF measured by the ABTS assay and expressed as percentage of antioxidant power (AOP) per mg of film.

The effect of LCNF and pre-treatment on AOP was statistically significant (*p* < 0.05). In turn, no remarkable differences were detected for the type of raw material (*p* > 0.05).

An increase in AOP was observed when the concentration of LCNF increased, exhibiting the greatest AOP value at 7% MP-LCNF, consisting of a rise from 1.1% AOP/mg registered in only-PVA film to 3.21% AOP/mg for this MP-LCNF formulation.

As shown in Figure 5, the films formulated with mechanical LCNF showed higher AOP values than those presented by TEMPO LCNF, though for 2% eggplant LCNF, it was less apparent. This was because the mechanical pre-treatment does not affect the oxidation state of the fibres, compared to TEMPO pre-treatment, preserving more of their antioxidant capacity [45]. The AOP of the LCNF was determined in previous research, in which a higher antioxidant capacity of the mechanical LCNF was also observed (Table S1).

### 3.6. Water Permeability

The water vapour permeability of PVA and 7% PVA–LCNF films was evaluated. Films with 7% of LCNF were selected for this test based on the good results obtained for other parameters previously shown.

**Figure 6:**
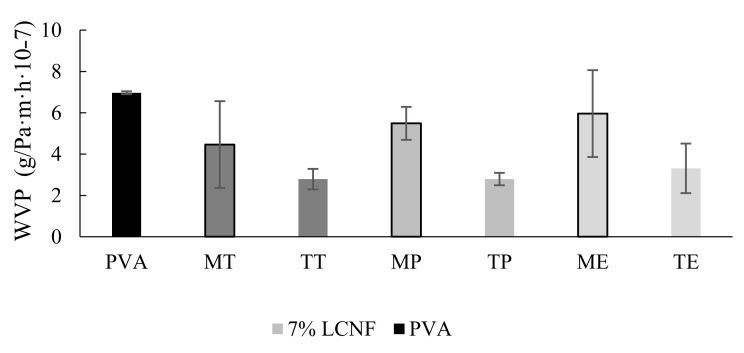
Water vapor permeability (WVP) of PVA films, PVA films containing 7% mechanical LCNF from tomato (MT), pepper (MP) and eggplant (ME) and PVA films containing 7% TEMPO LCNF from tomato (TT), pepper (TP) and eggplant (TE).

Figure 6 shows that the addition of 7% LCNF to the polymeric matrix decreased the water vapour permeability (WVP) with respect to only-PVA films, though values were not statistically different (*p* > 0.05). This apparent reduction could be due to the network formed between PVA and LCNF via hydrogen bonds, which reduces the free space in the PVA polymeric matrix, making it more difficult for water vapour to pass through the film [46].

Pre-treatment statistically influenced the WVP of films (*p* < 0.05). In this respect, films with TEMPO LCNF showed a lower WVP value as compared to mechanical LCNF. This reduction could be caused by the partial loss of lignin due to the oxidation process related to TEMPO pre-treatment. Lignin could hinder the formation of hydrogen bonds between the polymer matrix and the LCNF, resulting in a film with lower WVP, hence, the partial elimination of lignin in TEMPO would lead to better water vapour barrier properties [47].

### 3.7. Optical Properties

The transparency and UV barrier of only-PVA and PVA–LCNF films were determined by measuring the light transmittance in the UV and Visible range (200–800 nm).

The effect of LCNF on UV barrier and transparency was statistically significant (*p* < 0.05). This was not the case for the type of raw material and the pre-treatment, for which the differences were not significant (*p* > 0.05).

The optical transmittance of the films is highly dependent on the dispersion of LCNF in the PVA polymer matrix.

**Figure 7:**
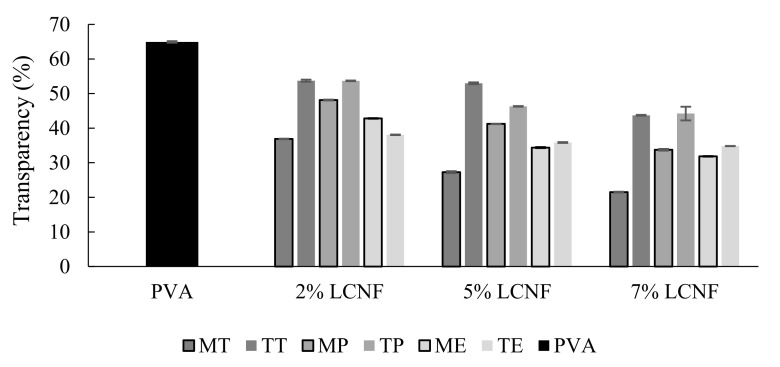
Transparency of PVA film, PVA films containing mechanical LCNF from tomato (MT), pepper (MP) and eggplant (ME) and PVA containing TEMPO LCNF from tomato (TT), pepper (TP) and eggplant (TE).

As can be seen in Figure 7, films with LCNF had lower transparency than those shown by the film with only PVA. Films became opaquer with increasing LCNF content, with transparency values decreasing by 66.9% in 7% MT-LCNF films in comparison with only-PVA film. In general, except for the samples with 2% eggplant LCNF, the LCNF oxidized by TEMPO pre-treatment increased the transparency of films. This increase could be related to a better nanofibrillation yield of these samples, which led to a higher dispersion in the polymeric matrix, thereby increasing light transmittance. [23].

**Figure 8:**
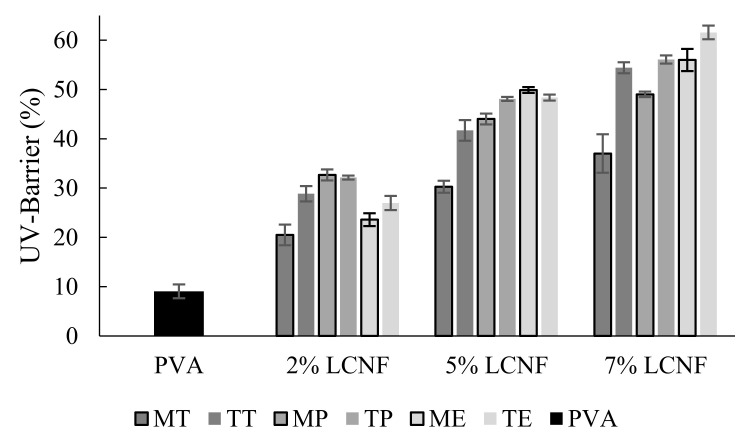
UV barrier of PVA film, PVA containing mechanical LCNF from tomato (MT), pepper (MP) and eggplant (T, MP, ME) and PVA containing TEMPO LCNF from tomato (TT), pepper (TP) and eggplant (TE).

By contrast, as shown in Figure 8, the only-PVA film exhibited lower UV-barrier capacity (9.98%) than LCNF films. The highest UV light blocking value (55.88%) was found in 7% ME-LCNF packages, while the lowest value (22.36%), excluding only-PVA film, corresponded with 2% MT-LCNF samples. This increasing trend in the capacity of blocking UV light as LCNF content rises can be clearly seen in Figure 8. However, the pre-treatment did not show any clear effect on this property and some results were contradictory. As indicated above, the optical transmittance of the films is highly dependent on the dispersion of nanofibres (i.e., LCNF) in the PVA polymer matrix. The same phenomenon could explain the increased UV light blocking of films with LCNF.

## 4. Conclusions

In this study, lignocellulose nanofibres (LCNF) obtained from agricultural waste of tomato, pepper and eggplant crops were proved to be a suitable reinforcement of PVA films, improving several properties of the film such as thermal resistance, water vapor permeability and mechanical parameters, among others, in addition to conferring antioxidant capacity, which was not present in the non-reinforced film. In particular, films formulated with 7% of LCNF from eggplant crop residues showed better results in terms of Young´s modulus, elongation at break, thermal stability and UV barrier. Results demonstrated that agri-food residues can be satisfactorily valorised, generating innovative nanomaterials able to enhance food packaging systems from a more sustainable and eco-friendly perspective. Nonetheless, further research is needed to assess and validate the performance and safety of the LCNF-reinforced PVA films on actual foods, simulating typical distribution and storage conditions.

## Figures and Tables

**Table 1:** Composition, pre-treatment and codification of the different film formulations.

Raw Material	Pre-Treatment	Codification	PVA–LCNF Concentration (%)	Codification
Polyvinyl alcohol		PVA	100	PVA
Tomato plant	Mechanical	Mechanical tomato (MT)	98:2	2% MT-LCNF
			95:5	5% MT-LCNF
			93:7	7% MT-LCNF
	TEMPO-mediated oxidation	TEMPO tomato (TT)	98:2	2% TT-LCNF
			95:5	5% TT-LCNF
			93:7	7% TT-LCNF
Pepper plant	Mechanical	Mechanical pepper (MP)	98:2	2% MP-LCNF
			95:5	5% MP-LCNF
			93:7	7% MP-LCNF
	TEMPO-mediated oxidation	TEMPO pepper (TP)	98:2	2% TP-LCNF
			95:5	5% TP-LCNF
			93:7	7% TP-LCNF
Eggplant plant	Mechanical	Mechanical eggplant (ME)	98:2	2% ME-LCNF
			95:5	5% ME-LCNF
			93:7	7% ME-LCNF
	TEMPO-mediated oxidation	TEMPO eggplant (TE)	98:2	2% TE-LCNF
			95:5	5% TE-LCNF
			93:7	7% TE-LCNF

**Table 2:** Degradation temperatures from TGA of PVA films, PVA films containing mechanical and PVA films containing TEMPO LCNF from tomato, pepper and eggplant.

Formulation		LCNF Concentration (%)	T_onset_ (°C) ^a^	T_max_ (°C) ^b^
PVA			210.0	250.32
Mechanical pre-treatment	Tomato	2	213.5	256.14
		5	217.4	262.88
		7	217.6	269.55
	Pepper	2	209.7	261.87
		5	212.4	266.14
		7	217.6	271.74
	Eggplant	2	216.0	263.76
		5	221.5	268.01
		7	231.3	277.36
TEMPO pre-treatment	Tomato	2	214.0	259.19
		5	218.1	262.06
		7	221.2	269.14
	Pepper	2	208.7	256.11
		5	211.2	264.33
		7	224.1	269.73
	Eggplant	2	218.5	258.20
		5	220.5	261.76
		7	223.6	264.14

^a^ T_onset_ (°C) is the temperature at which degradation of the material begins, which is calculated as the point of intersection of the starting mass baseline and the tangent to the TGA curve at the point of maximum gradient. ^b^ T_max_ (°C) is the maximum degradation rate temperature calculated as the derivative of TGA (DTG).

## Data Availability

The data presented in this study are available on request from the first author.

## Supplementary Materials

The following are available online at https://www.mdpi.com/article/10.3390/foods10123043/s1, Figure S1: TGA and DTG analysis of PVA films and PVA-LCNF films containing mechanical LCNF from tomato (MT), pepper (MP) and eggplant (ME) and PVA containing TEMPO LCNF from tomato (TT), pepper (TP) and eggplant (TE). Table S1: Antioxidant power (AOP) of mechanical and TEMPO-LCNF from tomato, pepper, and eggplant.

## References

[1] Han J.W., Ruiz-Garcia L., Qian J.P., Yang X.T. (2018). Food Packaging: A Comprehensive Review and Future Trends. Compr. Rev. Food Sci. Food Saf..

[2] Sid S., Mor R.S., Kishore A., Sharanagat V.S. (2021). Bio-sourced polymers as alternatives to conventional food packaging materials: A review. Trends Food Sci. Technol..

[3] Asgher M., Qamar S.A., Bilal M., Iqbal H.M.N. (2020). Bio-based active food packaging materials: Sustainable alternative to conventional petrochemical-based packaging materials. Food Res. Int..

[4] Plastic Europe An Analysis of European Plastics Production, Demand and Waste Data. https://www.mdpi.com/journal/foods/instructions#preparation.

[5] Ahmed T., Shahid M., Azeem F., Rasul I., Shah A.A., Noman M., Hameed A., Manzoor N., Manzoor I., Muhammad S. (2018). Biodegradation of plastics: Current scenario and future prospects for environmental safety. Environ. Sci. Pollut. Res..

[6] Bilal M., Gul I., Basharat A., Qamar S.A. (2021). Polysaccharides-based bio-nanostructures and their potential food applications. Int. J. Biol. Macromol..

[7] Rhim J.W., Park H.M., Ha C.S. (2013). Bio-nanocomposites for food packaging applications. Prog. Polym. Sci..

[8] Othman S.H. (2014). Bio-nanocomposite Materials for Food Packaging Applications: Types of Biopolymer and Nano-sized Filler. Agric. Agric. Sci. Procedia.

[9] De Azeredo H.M.C. (2009). Nanocomposites for food packaging applications. Food Res. Int..

[10] Zhang H., Wang Q., Li L. (2009). Dehydration of water-plasticized poly(vinyl alcohol) systems: Particular behavior of isothermal mass transfer. Polym. Int..

[11] Rudra R., Kumar V., Kundu P.P. (2015). Acid catalysed cross-linking of poly vinyl alcohol (PVA) by glutaraldehyde: Effect of crosslink density on the characteristics of PVA membranes used in single chambered microbial fuel cells. RSC Adv..

[12] Paralikar S.A., Simonsen J., Lombardi J. (2008). Poly(vinyl alcohol)/cellulose nanocrystal barrier membranes. J. Membr. Sci..

[13] Musetti A., Paderni K., Fabbri P., Pulvirenti A., Al-Moghazy M., Fava P. (2014). Poly(vinyl alcohol)-Based Film Potentially Suitable for Antimicrobial Packaging Applications. J. Food Sci..

[14] Tripathi S., Mehrotra G.K., Dutta P.K. (2009). Physicochemical and bioactivity of cross-linked chitosan–PVA film for food packaging applications. Int. J. Biol. Macromol..

[15] Gohil J.M., Bhattacharya A., Ray P. (2006). Studies on the crosslinking of poly(vinyl alcohol). J. Polym. Res..

[16] Lu J., Wang T., Drzal L.T. (2008). Preparation and properties of microfibrillated cellulose polyvinyl alcohol composite materials. Compos. Part. A Appl. Sci. Manuf..

[17] Liu Y., Ahmed S., Sameen D.E., Wang Y., Lu R., Dai J., Li S., Qin W. (2021). A review of cellulose and its derivatives in biopolymer-based for food packaging application. Trends Food Sci. Technol..

[18] Amara C., El Mahdi A., Medimagh R., Khwaldia K. (2021). Nanocellulose-based composites for packaging applications. Curr. Opin. Green Sustain. Chem..

[19] Dubief D., Samain E., Dufresne A. (1999). Polysaccharide Microcrystals Reinforced Amorphous Poly(β-hydroxyoctanoate) Nanocomposite Materials. Macromolecules.

[20] Sánchez-Gutiérrez M., Bascón-Villegas I., Espinosa E., Carrasco E., Pérez-Rodríguez F., Rodríguez A. (2021). Cellulose Nanofibers from Olive Tree Pruning as Food Packaging Additive of a Biodegradable Film. Foods.

[21] Ministerio de Agricultura, Pesca y Alimentación Cifras del Sector de Frutas y Hortalizas. https://www.mapa.gob.es/es/agricultura/temas/producciones-agricolas/cifrasdelsectorfyhactualizado2020definitivo-junio2021_tcm30-563965.pdf.

[22] Sánchez-Gutiérrez M., Espinosa E., Bascón-Villegas I., Pérez-Rodríguez F., Carrasco E., Rodríguez A. (2020). Production of Cellulose Nanofibers from Olive Tree Harvest—A Residue with Wide Applications. Agronomy.

[23] Bascón-Villegas I., Espinosa E., Sánchez R., Tarrés Q., Pérez-Rodríguez F., Rodríguez A. (2020). Horticultural Plant Residues as New Source for Lignocellulose Nanofibers Isolation: Application on the Recycling Paperboard Process. Molecules.

[24] Espinosa E., Sánchez R., Otero R., Domínguez-Robles J., Rodríguez A. (2017). A comparative study of the suitability of different cereal straws for lignocellulose nanofibers isolation. Int. J. Biol. Macromol..

[25] Besbes I., Alila S., Boufi S. (2011). Nanofibrillated cellulose from TEMPO-oxidized eucalyptus fibres: Effect of the carboxyl content. Carbohydr. Polym..

[26] Espinosa E., Sánchez R., González Z., Domínguez-Robles J., Ferrari B., Rodríguez A. (2017). Rapidly growing vegetables as new sources for lignocellulose nanofibre isolation: Physicochemical, thermal and rheological characterisation. Carbohydr. Polym..

[27] Sarwar M.S., Niazi M.B.K., Jahan Z., Ahmad T., Hussain A. (2018). Preparation and characterization of PVA/nanocellulose/Ag nanocomposite films for antimicrobial food packaging. Carbohydr. Polym..

[28] Re R., Pellegrini N., Proteggente A., Pannala A., Yang M., Rice-Evans C. (1999). Antioxidant activity applying an improved ABTS radical cation decolorization assay. Free Radic. Biol. Med..

[29] Nesic A., Seslija S.I. (2017). The influence of nanofillers on physical–chemical properties of polysaccharide-based film intended for food packaging. Food Packag..

[30] Markus Schmid K.M., Deeth H.C., Bansal N. (2019). Chapter 11—Whey Protein-Based Packaging Films and Coatings. En Whey Proteins from Milk to Medicine.

[31] Singh S., Gaikwad K.K., Lee Y.S. (2018). Antimicrobial and antioxidant properties of polyvinyl alcohol bio composite films containing seaweed extracted cellulose nano-crystal and basil leaves extract. Int. J. Biol. Macromol..

[32] Pereda M., Dufresne A., Aranguren M.I., Marcovich N.E. (2014). Polyelectrolyte films based on chitosan/olive oil and reinforced with cellulose nanocrystals. Carbohydr. Polym..

[33] Assender H.E., Windle A.H. (1998). Crystallinity in poly(vinyl alcohol) 1. An X-ray diffraction study of atactic PVOH. Polymer.

[34] Popescu M.-C. (2017). Structure and sorption properties of CNC reinforced PVA films. Int. J. Biol. Macromol..

[35] Lee H., You J., Jin H.-J., Kwak H.W. (2020). Chemical and physical reinforcement behavior of dialdehyde nanocellulose in PVA composite film: A comparison of nanofiber and nanocrystal. Carbohydr. Polym..

[36] Niu X., Liu Y., Fang G., Huang C., Rojas O.J., Pan H. (2018). Highly Transparent, Strong, and Flexible Films with Modified Cellulose Nanofiber Bearing UV Shielding Property. Biomacromolecules.

[37] Othman S.H., Nordin N., Azman N.A.A., Tawakkal I.S.M.A., Basha R.K. (2021). Effects of nanocellulose fiber and thymol on mechanical, thermal, and barrier properties of corn starch films. Int. J. Biol. Macromol..

[38] Nair S.S., Yan N. (2015). Effect of high residual lignin on the thermal stability of nanofibrils and its enhanced mechanical performance in aqueous environments. Cellulose.

[39] Chen Y., Fan D., Han Y., Lyu S., Lu Y., Li G., Jiang F., Wang S. (2018). Effect of high residual lignin on the properties of cellulose nanofibrils/films. Cellulose.

[40] Ingrao C., Bacenetti J., Bezama A., Blok V., Goglio P., Koukios E.G., Lindner M., Nemecek T., Siracusa V., Zabaniotou A. (2018). The potential roles of bio-economy in the transition to equitable, sustainable, post fossil-carbon societies: Findings from this virtual special issue. J. Clean. Prod..

[41] Mathijs E., Brunori G., Carus M., Griffon M., Last L., Barna K. (2015). Sustainable Agriculture, Forestry and Fisheries in the Bioeconomy. A Challenge for Europe. 4th SCAR Foresight Exercise.

[42] Mandal A., Chakrabarty D. (2014). Studies on the mechanical, thermal, morphological and barrier properties of nanocomposites based on poly(vinyl alcohol) and nanocellulose from sugarcane bagasse. J. Ind. Eng. Chem..

[43] Pereira V.A., de Arruda I.N.Q., Stefani R. (2015). Active chitosan/PVA films with anthocyanins from *Brassica oleraceae* (Red Cabbage) as Time–Temperature Indicators for application in intelligent food packaging. Food Hydrocoll..

[44] Wang X., Bian H., Ni S., Sun S., Jiao L., Dai H. (2020). BNNS/PVA bilayer composite film with multiple-improved properties by the synergistic actions of cellulose nanofibrils and lignin nanoparticles. Int. J. Biol. Macromol..

[45] Thá E.L., Matos M., Avelino F., Lomonaco D., Rodrigues-Souza I., Gagosian V.S.C., Cestari M.M., Magalhães W.L.E., Leme D.M. (2021). Safety aspects of kraft lignin fractions: Discussions on the in chemico antioxidant activity and the induction of oxidative stress on a cell-based in vitro model. Int. J. Biol. Macromol..

[46] Naidu D.S., John M.J. (2021). *Cellulose nanofibrils* reinforced xylan-alginate composites: Mechanical, thermal and barrier properties. Int. J. Biol. Macromol..

[47] Spence K.L., Venditti R.A., Rojas O.J., Habibi Y., Pawlak J.J. (2010). The effect of chemical composition on microfibrillar cellulose films from wood pulps: Water interactions and physical properties for packaging applications. Cellulose.

