# Supplementary material for: Lignocellulose Nanofibre Obtained from Agricultural Wastes of Tomato, Pepper and Eggplants Improves the Performance of Films of Polyvinyl Alcohol (PVA) for Food Packaging
Source: Foods. 2021 Dec 8;10(12):3043. doi: 10.3390/foods10123043 (PMC8700978; doi:10.3390/foods10123043)
Supplement: Supplementary file 1 [file foods-10-03043-s001.zip › foods-1439345-supplementary.pdf]

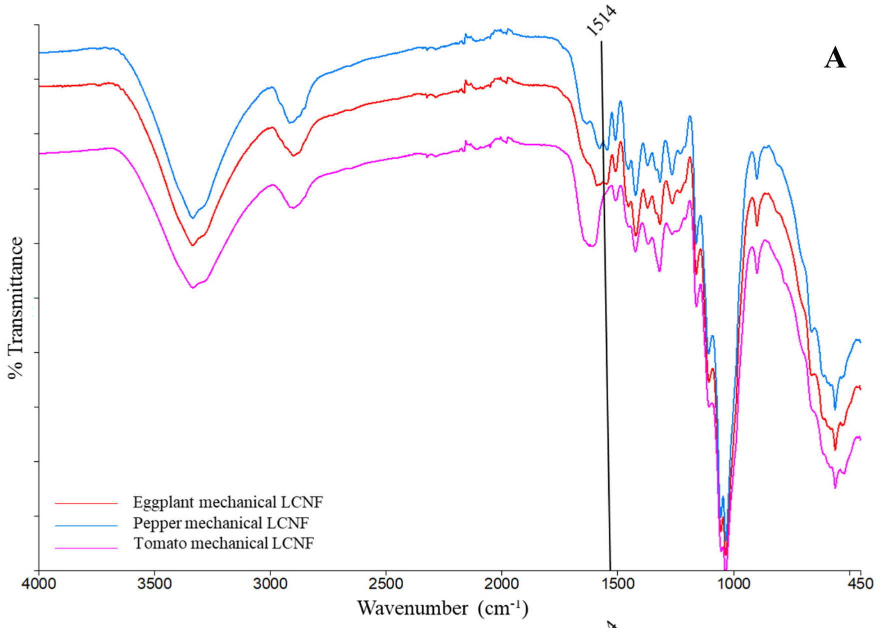

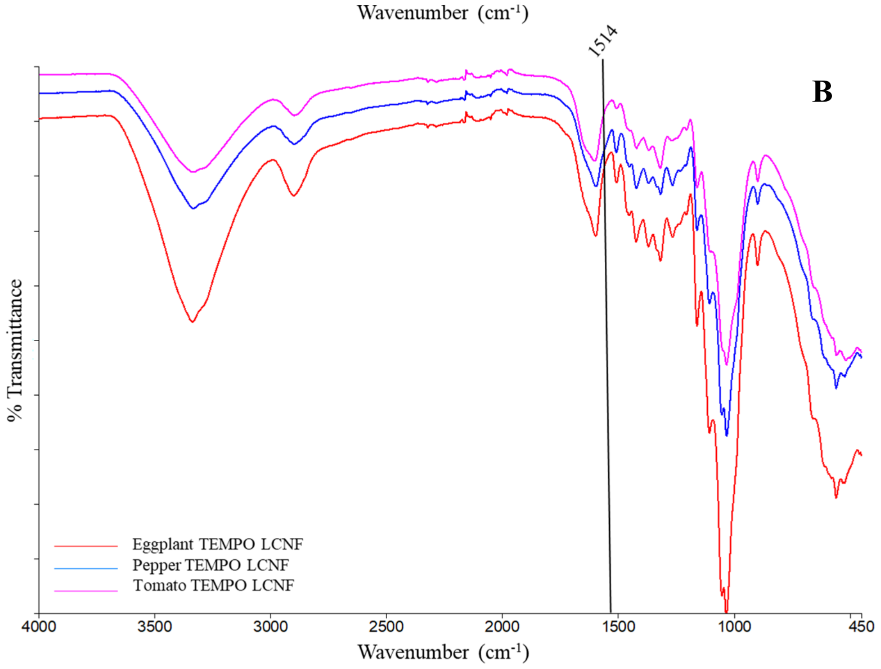

Figure S1. FT-IR spectra of mechanical-LCNF (A), and TEMPO-LCNF (B) from tomato, pepper, and eggplant.

Table S1. Antioxidant power (AOP) of mechanical and TEMPO-LCNF from tomato, pepper, and eggplant.

**Sample**	**Pre-treatment**	**AOP (%) ± SD**
Tomato	Mechanical	25.16 ± 0.09
	TEMPO	13.96 ± 0.19
Pepper	Mechanical	20.55 ± 0.06
	TEMPO	15.44 ± 0.12
Eggplant	Mechanical	26.64 ± 0.15
	TEMPO	13.93 ± 0.07
